# Metabolic abnormalities in pure vs. mixed struvite stone formers: A retrospective comparative analysis utilising large language models for data extraction

**DOI:** 10.1002/bco2.70072

**Published:** 2025-09-22

**Authors:** Juanita Velasquez Ospina, Ansh Bhatia, Archan Khandekar, Aravindh Rathinam, Glenn Austin, Jonathan Katz, Robert Marcovich, Hemendra N. Shah

**Affiliations:** ^1^ Desai Sethi Urology Institute University of Miami, Miller School of Medicine Miami Florida USA; ^2^ Department of Interventional Radiology University of Miami, Miller School of Medicine Miami Florida USA; ^3^ Louis C. Herring & Company Laboratory Orlando Florida USA

**Keywords:** calcium oxalate nephrolithiasis, medical management of stones, metabolic evaluation of stones, stone recurrence, struvite stones

## Abstract

**Objectives:**

To evaluate demographic characteristics and the prevalence of metabolic abnormalities in patients with pure struvite stones compared to those with mixed struvite and calcium oxalate stones.

**Methods:**

We retrospectively reviewed 3001 stone analyses performed at our institution between August 2019 and April 2024. Patients who had a 24‐hour urinary stone risk profile within six months of stone analysis were included. Exclusion criteria were a history of nephrolithiasis, bladder stones, neurogenic bladder or surgery for encrusted stent removal. Demographics, comorbidities and laboratory values were extracted using a HIPAA‐compliant pipeline enhanced with a large language model (LLM). Patients with pure struvite stones were compared to those with mixed calcium oxalate–struvite stones. Statistical analysis was performed using RStudio v4.1.3.

**Results:**

Forty‐one patients met the inclusion criteria: 21 with pure struvite stones and 20 with mixed stones. While pure struvite stones were more common in females, the difference was not statistically significant. Demographics and comorbidities were similar between groups. Patients with pure struvite stones showed lower urinary levels of oxalate, potassium, citrate and uric acid, though not statistically significant. However, supersaturation of brushite, calcium oxalate and sodium urate was significantly lower in the pure struvite group (P < 0.05). At least one metabolic abnormality was present in 90.5% of the pure struvite group and in all patients with mixed stones.

**Conclusions:**

Metabolic abnormalities are highly prevalent in both pure and mixed struvite stone formers. These findings support routine metabolic evaluation in patients with infection‐related stones to guide long‐term management.

## 1 INTRODUCTION

Nephrolithiasis incidence is increasing globally, with a lifetime prevalence reaching up to 19% in some populations.^1^ Among stone types, infection‐related stones account for approximately 7–8% of cases. They are predominantly composed of magnesium ammonium phosphate (struvite) or carbonate apatite^2^ and are commonly associated with urinary tract infections (UTIs) caused by urease‐producing bacteria such as *Proteus*, *Klebsiella*, *Pseudomonas* and *Staphylococcus* species.^3^ Urease hydrolyses urea into ammonia and carbon dioxide, resulting in an alkaline urinary environment that promotes the formation of struvite and carbonate apatite crystals.^2^, ^3^ If left untreated, infection stones can cause recurrent UTIs, renal impairment and even death.^4^ Notably, the recurrence rate of pure struvite stones is 41.6%, and for struvite in combination with other stone types is 40.1%.^5^

Although metabolic evaluation, including 24‐hour urine collection, is recommended for patients with nephrolithiasis, its role in infection stone formers remains controversial. Early studies in the 1980s recommended routine metabolic workup, citing the potential presence of underlying metabolic abnormalities in struvite stones.^6^, ^7^ However, in one of those studies, only one of 33 patients had pure struvite stones. Lingeman et al. later questioned this recommendation, pointing out that results may have been confounded by the inclusion of mixed‐composition stones rather than pure struvite.^8^ As a result, the hypothesis that struvite stones lack underlying metabolic abnormalities was likely accepted by urologists worldwide, which may explain the paucity of related publications over the past two decades.

Recently, a few retrospective studies have revived interest in this topic. While some studies report metabolic abnormalities in up to 71% of infection stone formers, others have found rates of metabolic abnormalities as low as 10.5%.^2^, ^4^, ^9^, ^10^ The 2019 American Urological Association (AUA) guidelines recommend 24‐hour urine testing in high‐risk or recurrent stone formers, which includes those with recurrent UTIs.^11^ However, they do not specifically address the role of metabolic evaluation in patients with struvite stones. The 2025 European Association of Urology (EAU) guidelines go further, recommending metabolic evaluation after stone removal and infection control in patients with mixed struvite stones.^12^ In this study, we aimed to assess the demographic characteristics and prevalence of metabolic abnormalities in patients with pure struvite stones compared to those with mixed struvite and calcium oxalate (CaOx) stones. We believe our findings contribute to the ongoing discussion on the clinical utility of metabolic workup in infection‐related nephrolithiasis and may help guide future strategies for preventing stone recurrence in struvite stones.

## 2 METHODS

At our institution, all patients undergoing medical or surgical treatment for nephrolithiasis routinely have their stone composition analysed. We retrospectively collected all stone analysis reports from August 2019 to April 2024. A HIPAA‐compliant method leveraging a large language model (LLM) (Claude 3.7 foundation model) was employed to extract stone composition data from PDF files of these reports. The LLM parsed unstructured documents to extract stone composition along with patient demographics, including age and sex. Additional information—such as medical comorbidities, laboratory values and 24‐hour urine profiles—was obtained from our university's electronic medical record system via Theator to ensure confidentiality. The 24‐hour urine profile result done within the first 6 months from the date of stone analysis was considered for this study. All extracted data were compiled into a structured Excel database, enabling detailed assessment of demographics, comorbidities, lab values, stone types and metabolic risk profiles. To ensure accuracy, a random sample of 15 patient records was manually reviewed by a co‐author (AK). Institutional Review Board approval was obtained (IRB number 20180511).

Statistically assuming the LLM was ≥95% accurate, to detect an error rate >5% with 95% confidence, a manual chart review of at least 60 charts with zero errors was needed—to be 95% confident the true error rate is <5%. Hence, a random sample of 60 patient records was manually reviewed by a co‐author (AK).

We then identified patients with either pure struvite stones or mixed struvite and CaOx stones. Inclusion was limited to patients with kidney or ureteric stones. Exclusion criteria included a history of nephrolithiasis, neurogenic bladder, or surgeries performed for the removal of retained encrusted stents. From the remaining cohort, we included only patients who had undergone 24‐hour urine metabolic testing within three months of their stone analysis and who were not on any pharmacologic treatment for urolithiasis at the time. All patients included had a negative urine culture at the time of metabolic evaluation.

Collected variables included age, sex, body mass index (BMI), race/ethnicity and chronic comorbidities. All components of the 24‐hour urine profiles were included in the analysis. Prior stone history was excluded to focus on first‐time stone formers and avoid confounding by prior treatment or metabolic management. Continuous variables were assessed for normality using visual inspection and the Shapiro–Wilk test. Normally distributed variables were reported as mean ± standard deviation (SD), while non‐normally distributed variables were expressed as median with interquartile range (IQR). Comparisons between groups were made using unpaired t‐tests or Wilcoxon rank‐sum tests, as appropriate. All statistical analyses were performed using RStudio version 4.1.3 (RStudio Inc., MA, USA). We also reviewed literature on PubMed to look for all manuscripts evaluating metabolic anomalies in infectious stones (Table 1).

**TABLE 1:** Publications of metabolic abnormalities in infection stones^2^, ^4^, ^8^, ^9^, ^10^.

Authors (Year); Country of origin; Study type	Sample Size and Gender Distribution, n (female %)	Group(s) Analysed	Metabolic Abnormalities Per Group Analysed, n (%)	Specific Metabolic Abnormalities
Lingeman et al. (1995)^8^; USA; Retrospective cohort	21, (69%)	Pure struvite stones: MAP ± CA (**n = 14**)	2/14 (14.3%)	Hypercalciuria (14.3%)
		Mixed stones: MAP ± CA mixed with other crystalline materials (n = 7)	7/7 (100%)	Hypercalciuria (71.4%) hyperuricosuria (14.3%%)
Cicerello et al. (2016)^10^; Italy; Cross‐sectional	54, (61.1%)	Pure infected stones: Struvite ± CA (**n = 19**)	3/19 (15.8%)	Hypercalciuria (10%) hyperoxaluria (5%)
		Mixed infected stones: Struvite ± CA AND CaOx (n = 35)	Unspecified	Hypercalciuria (40%) hyperoxaluria (34%) hyperuricosuria (28%)
Iqbal et al. (2017)^4^; USA; Retrospective cohort	75^a^, (48%)	Pure struvite stones: 100% of MAP ± CA (**n = 7**)	4/7 (57%)	Hypercalciuria (10%) hyperoxaluria (5%) hyperuricosuria (29%) hypocitraturia (14%)
		Mixed struvite stones: Any amount of struvite with other stone compositions (n = 32)	26/32 (81%)	Hypercalciuria (38%) hyperoxaluria (16%) hyperuricosuria (38%) hypocitraturia (41%) gouty diathesis (13%)
Danilovic (2021)^9^; Brazil; Retrospective cohort	67, (83.6%)	Pure MAP stones (**n = 28**)	20/28 (71.4%)	Hypercalciuria (7.1%) hypocitraturia (65.2%) hypernatriuria (13%)
		CaOx stones (n = 39)	26/39 (66.6%)	Hypercalciuria (10.3%) hyperuricosuria (5.1%) hypocitraturia (59%) hypernatriuria (10.3%)
Popovtzer et al. (2022)^2^; USA & Israel; Retrospective cohort	154^b^, (65.6%)	MAP stones: composed of >50% MAP (**n = 50**)	Unspecified	Hypercalciuria (38%); hypocitraturia (71%)
		CA stones: composed of >30% CA (n = 49)	Unspecified	Hypercalciuria (32%); hypocitraturia (66%)

(MAP‐Magnesium ammonium phosphate; CA‐Carbonate apatite; CaOx‐Calcium oxalate).

^a^ Of the 75 patients, only 39 had metabolic evaluation available.

^b^ Not all patients had 24‐hour urine collection available, but no numbers were specified.

## 3 RESULTS

Out of 3001 stone analyses conducted during the study period, data for 24‐hour urine profiles performed within six months of the stone analysis were available for 659 patients after removing duplicates. Among these, 41 patients with kidney or ureteral stones and no prior history of nephrolithiasis were identified to have a struvite component in their stones and met the inclusion criteria for this study. Of these, 21 patients had pure struvite stones, while the remaining 20 had mixed CaOx–struvite stones.

There were no statistically significant differences between the groups in demographic characteristics, including age, BMI, gender, and race (Table 2). While pure struvite stones were more commonly observed in females (80% vs. 50%), this difference did not reach statistical significance. Comorbidities such as diabetes mellitus, hypertension, gout, and hyperlipidemia were similarly distributed across both groups. Metabolic evaluations revealed lower mean urinary levels of oxalate, potassium, citrate, and uric acid in the pure struvite group, but these differences were not statistically significant (Table 3). However, supersaturation levels of brushite (p = 0.009), CaOx (p = 0.02), and sodium urate (p = 0.03) were significantly lower in the pure struvite group compared to the mixed group.

**TABLE 2:** Clinical and demographic characteristics.

Variable	Pure struvite stone	Mixed Struvite‐Calcium oxalate stone	P value
N	21	20	
Age (year)	56.6 ± 14.5	52.3 ± 18.5	0.41
BMI	29.0 ± 7.2	29.0 ± 6.5	0.99
BMI > 40	2 (9.52%)	1 (5%)	1
Sex			0.056
Male	4 (19%)	10 (50%)	
Female	17 (81%)	10 (50%)	
Race			
White	5	2	
Black	1	2	0.26
Hispanic	14	14	
Other/NA	1	2	
Associated medical co‐morbidities			
Diabetes mellitus	7 (33.3%)	4 (20%)	0.45
Hypertension	11 (52.38%)	8 (40%)	0.67
Gout	0	0	1
Hyperlipidaemia	8 (38%)	9 (45%)	0.70

(BMI‐ body mass index; NA‐ not available).

**TABLE 3:** 24‐hour urinary metabolic abnormalities.

Parameters	Normal values	Pure struvite stone	Mixed Struvite ‐Calcium oxalate stone	P value
		21	20	
Urine volume, *L/day*	>2.00	1.9 ± 0.7	2.02 ± 0.9	0.64
Calcium, urine total, *mg/day*	<250.0	155.7 ± 84.8	180.8 ± 89.7	0.43
Urine PH	5.5–7.0	6.4 ± 0.7	6.4 ± 0.6	0.89
Citrate in urine, *mg/day*	>320	358.9 ± 239.0	520.1 ± 432.2	0.31
Potassium in urine, *mEq/day*	19–135	47.4 ± 23.4	60.6 ± 26.4	0.16
Oxalate in urine, *mg/day*	<45	26.5 ± 18.0	39.9 ± 25.2	0.11
Uric Acid in urine, *mg/day*	<700	555.5 ± 259.6	724.2 ± 413.6	0.20
Magnesium in urine, *mg/day*	>60.0	82.5 ± 37.9	106.6 ± 72.9	0.28
Sodium in urine, *mg/day*	<200	132.1 ± 91.0	164.6 ± 103.1	0.34
Sulfate in urine, *mmol/day*	<30	16.6 ± 14.7	22.9 ± 25.9	0.42
Phosphorus in urine, *mg/day*	<1100	744.4 ± 418.0	1002.5 ± 554.3	0.10
**Super saturation**				
Brushite	<2	1.1 ± 0.5	2.5 ± 1.10	**0.009**
CaOx	<2	1.0 ± 0.8	1.9 ± 1.0	**0.02**
Sodium urate	<2	1.3 ± 1.1	3.9 ± 3.5	**0.03**
Uric acid	<2	1.6 ± 1.7	1.4 ± 1.7	0.74
**Percentage of patient with metabolic abnormality**				
urine volume < 1.5 lit	<1.5 L	29.4%	35.7%	1
Urine PH > 7	< 7	11.8%	26.7%	0.38
hypercalciuria	>250	17.6%	21.4%	1
hyperoxaluria	>45	47.6%	50%	1
hyperuricosuria	>700	31.76%	28.6%	0.68
hypocitraturia	<320	58.8%	64.3%	1
High uric acid supersaturation	>2	44.4%	27.2%	0.65
High calcium oxalate saturation	>2	11.1%	45.5%	0.15
High sodium urate saturation	>2	41.2%	27.2%	0.64
High brushite saturation	>2	47.6%	63.6%	0.71

Both groups exhibited a high prevalence of metabolic abnormalities, with at least one being identified in 90.5% (19/21) of patients in the pure struvite group and 100% (20/20) in the mixed group. Elevated urine pH (>7) was seen in 11.8% of pure struvite patients and 26.7% of those with mixed stones. Hypercalciuria was present in 17.6% and 21.4%, and hyperuricosuria in 31.7% and 28.6% of the pure and mixed groups, respectively. Hyperoxaluria affected 50% of patients in both groups. Hypocitraturia emerged as the most common abnormality, observed in 58.8% of the pure struvite group and 64.3% of the mixed group. However, none of these differences reached statistical significance (Table 3).

## 4 DISCUSSION

Given the variability in reported metabolic abnormality rates, and the small sample sizes in prior studies, the role of metabolic evaluation in struvite stone formers remains unclear, with only four previous studies specifically comparing pure struvite stone formers to other stone types (Table 1).^4^, ^8^, ^9^, ^10^ While surgical intervention and antibiotic therapy remain the cornerstone treatment for infection stones, the potential benefit of metabolic evaluation and management remains unclear.^13^ Although the overall prevalence of these stones has declined, they continue to be associated with significant morbidity and mortality, and recurrence remains a major concern.^2^, ^4^, ^14^ Long‐term recurrence rates are high, with some studies reporting up to 52% at 10 years and struvite‐specific recurrence reaching 41.6%.^5^, ^15^ This underscores the importance of identifying modifiable risk factors to prevent future stone formation. We found a high prevalence of metabolic abnormalities in our cohort, highlighting an opportunity for targeted medical management to potentially reduce recurrence risk.

We found similar demographics and clinical characteristics between groups, with factors such as age, BMI, race, and associated medical comorbidities being comparable. Our struvite group had a female preponderance, congruent with studies revealing a higher proportion of struvite stones in this gender, although this did not reach statistical significance.^16^ Severe obesity, diabetes mellitus and hypertension prevalence was higher in the pure struvite group. Hyperlipidemia, on the other hand, was the only metabolic trait higher in the mixed stone formers group. Our findings are congruent with those of Torricelli et al. who noted a link between dyslipidemia and kidney stone risk that was independent of other components of metabolic syndrome such as diabetes and obesity.^17^ Even though the differences in the incidence of associated medical co‐morbidities did not meet statistical significance, the higher prevalence of metabolic comorbidities in the struvite stone group is somewhat unexpected, given that metabolic syndrome is a well‐known risk factor for CaOx stone formation.^18^, ^19^, ^20^, ^21^ Nevertheless, comorbidities have been previously described in infection stone formers, with one study identifying obesity in 24% of their cohort, diabetes mellitus in 16.9%, hypertension in 33.8% and hyperlipidemia in 31.8%.^2^ This could suggest that these subsets of patients are not as different as previously thought.

The overall rate of metabolic abnormalities was high in both groups, with no statistically significant differences identified, which is consistent with prior findings reported by Iqbal et al. and Danilovic et al., who found metabolic abnormalities in 57% and 71.4% of their pure struvite groups, respectively.^4^, ^9^ Conversely, older studies such as those by Cicerello et al. and Lingeman et al. reported much lower rates (15.8% and 14.3%, respectively), possibly reflecting evolving patient populations in more recent studies^8^, ^10^.

As expected, the levels of urinary calcium, oxalate, uric acid and sodium were higher in our mixed stone group; however, the differences were not statistically significant. Urinary calcium levels were 155 ± 84.8 in our pure struvite group compared to 180.8 ± 89.7 in the mixed group, and in parallel, we found higher rates of hypercalciuria in the latter group (21.4%). This metabolic abnormality is a well‐recognised risk factor for stone formation, with authors reporting varying prevalence of hypercalciuria ranging from 7.1% to 14.3% in their pure struvite stone groups.^4^, ^8^, ^9^, ^10^ Even though similar, we found the prevalence of hypercalciuria to be slightly higher in our struvite stone formers. More recent studies have reported even higher rates of hypercalciuria in infection stones; for example, a multicentre study by Popovtzer and colleagues found rates of 38% and 32% in magnesium ammonium phosphate and carbonate apatite stones, respectively, including patients from the US and Israel, though their larger sample size and geographic variability could contribute to their higher rates.^2^

Citrate plays a critical role in preventing stone formation by binding urinary calcium and inhibiting crystallisation. Hypocitraturia, therefore, is a well‐known risk factor for nephrolithiasis.^4^, ^22^ A proposed mechanism for hypocitraturia in infection stones is bacterial degradation of urinary citrate via citrate lyase.^23^ In our study, urinary citrate levels were lower in the pure struvite group compared to the mixed group, though this difference was not statistically significant (p = 0.31). Hypocitraturia was prevalent in both groups of our study, similar to findings reported by Danilovic et al. and Popovtzer et al. (65.2% and 71%, respectively), while Iqbal et al. reported a much lower prevalence, likely due to their small sample size (n = 7 in their pure struvite group).^2^, ^4^, ^9^ The high prevalence in our cohort and others could suggest some metabolic overlap; however, in our pure struvite group, bacteriuria remains a possible explanation that cannot be overlooked.

Hyperoxaluria and hyperuricosuria were also observed in 50% and 31.76% of our pure struvite group, respectively, with the latter being more prevalent than in the mixed group. However, these differences were not statistically significant. Other studies have similarly found no significant differences in these parameters across stone types.^4^, ^9^

Supersaturation is considered a key step in stone formation.^24^, ^25^, ^26^, ^27^ In our analysis, supersaturation levels for brushite, CaOx, and sodium urate were significantly higher in the mixed group, which aligns with their calcium‐based stone composition.^26^, ^28^ Urinary pH, another critical factor in stone formation, was similar between groups (6.4 ± 0.7 vs. 6.4 ± 0.6). Although elevated urinary pH (>7.1) facilitates struvite formation, this alone may not fully account for stone development.^4^, ^9^, ^29^

Limitations of this study include its small sample size and retrospective design, which may limit generalisability and introduce selection bias. We also lacked data on anatomic abnormalities that could influence stone formation. Additionally, the exclusion of patients with a prior history of nephrolithiasis may limit the applicability of our findings to recurrent stone formers. Despite these limitations, our study adds to the sparse literature by directly comparing metabolic profiles between pure struvite and mixed struvite‐CaOx stone formers. Most prior studies focus on the infectious causes of struvite stones, but few examine whether metabolic differences exist between subtypes. Our findings provide insight into the metabolic profiles of infection stone formers, suggest that both groups may benefit from similar metabolic evaluation, and highlight the need for further research to determine whether targeted interventions can improve long‐term outcomes.

## 5 CONCLUSION

There is an overall high rate of metabolic abnormalities in both pure struvite and mixed struvite‐CaOx stone formers, with few significant differences in demographics and patient characteristics. Our findings support the need for metabolic assessment in all stone formers, including those with infection stones.

## AUTHOR CONTRIBUTIONS

J.V.O. contributed to data collection/management and manuscript writing/editing. A.B. was involved in protocol/project development, data collection/management, data analysis and manuscript editing. A.K. contributed to data collection/management, data analysis and manuscript editing. A.R. was involved in protocol/project development and manuscript writing/editing. G.A. contributed to data collection/management, data analysis and manuscript editing. J.K., R.M. and H.N.S. were involved in protocol/project development and manuscript writing/editing. All authors critically reviewed and approved the final version of the manuscript.

## CONFLICT OF INTEREST STATEMENT

All authors have completed the ICMJE conflicts of interest disclosure form. No conflicts of interest were reported by any author, including financial or non‐financial relationships such as funding, consulting, royalties, honoraria, travel support, stock ownership, patents, or leadership roles.

## Data Availability

All data supporting the findings of this study are available within the manuscript and its Supplementary Information.
